# Medical and Physical Intelligence

**Published:** 1805-01-01

**Authors:** 


### MEDICAL AND PHYSICAL INTELLIGENCE.

### QUERIES RELATING TO THE NATURAL HISTORY OF SYPHILIS.

Mr. BLAIR, surgeon of the Lock Hospital, &c. has been lately engaged in adding some notes to a translation of the celebrated Dr. Girtanner's Treatise upon the Venereal Disease; in which is contained a more elaborate and complete history of its origin and progress, than we have seen in any other publication. Many of our readers may not know that Dr. Girtanner has taken great pains to establish the common opinion (which Astruc espoused) of syphilis having been originally imported into Europe from America, by the companions of Columbus, in the year 1493.

But, Mr. Blair is said to have long since entertained an opposite opinion, and therefore has been induced to examine all the arguments on this subject, *de novo*. The general result of his enquiries (which he informs us were diligently prosecuted many years ago) is, that the venereal disease did not exist either in the American Islands or Continent, prior to the discoveries made by the Spaniards and others; and that there is no sufficient evidence of its transmission from Spain to Naples, and from thence to France, by the contending armies of Europe, who were at war about the year 1494.

Mr. Blair has communicated to us a few *queries* relative to this subject; which we willingly submit to the attention of our readers, in hopes that some correspondent may furnish him with a reply in our future Numbers.

1. What

1. What diseases among the ancients bore the nearest resemblance to syphilis, or the confirmed lues venerea, as it now exists in most parts of the globe?

2. Is there not good reason to conclude, that the yaws, or pians, (Frambœsia of Nosologists) prevailed in Asia, Africa, and America, at the precise time when the lues venerea was reputed to have made its first ravages in Europe?

3. Were not the yaws mistaken for the venereal disease by the early discoverers and historians of America; and has not this mistake given rise to the popular notion of syphilis being of American origin?

4. What epidemic disorders were predominant in Spain, Germany, Italy, &c. when the symptoms of lues venerea made so great a progress in Europe, and especially in those countries which were at war with France, before the conclusion of the 15th century?

5. Are there any positive historical proofs, founded on ocular evidence, of the genuine venereal disease existing among the sailors of Columbus, at the time of their return from Hispaniola to Portugal and Spain, A. D. 1493?

6. Was not the syphilitic virus conveyed to St. Domingo by the Spaniards, of whom Columbus says one hundred and sixty were infected in August, 1498; rather than that they received this infection from the native West Indians, as is generally affirmed by medical writers?

7. Have not the Europeans disseminated this direful complaint over the American continent, and among the South-Sea islanders, as well as in other newly discovered countries, where the syphilis was previously unknown?

8. Is there at present any nation in the world among whom this disease has not been experienced, or has but very recently been introduced; and can it now be eradicated entirely, or mitigated in its symptoms wherever it already exists?

---

Dr. Wollaston has discovered another new metal in crude platina, to which he has given the name of Rhodium. It is dissolved, together with the platina, in nitro-muriatic acid. From this solution, the platina being thrown down by sal-ammoniac, a plate of zinc precipitates all the other metals except iron. The black powder thrown down by the zinc is digested in very weak nitric acid, to dissolve any copper. The whole is then dissolved in nitro-muriatic-acid. Mix the solution with common salt, evaporated to dryness by a very gentle heat; wash the residuum with alcohol till the alcohol comes off colourless: what remains behind is a soda-muriat of rhodium. Dissolve it in water; add a cylinder of zinc; heat the black powder which is thus obtained, mixing it with borax. The powder becomes white, and acquires a metallic lustre. In this state it is pure rhodium. The name was given in consequence of the fine red solutions which it makes with acids. It is infusible; its specific gravity is 11. It is not precipitated by sal-ammoniac, common salt, prussiat of potash, or hydro-sulphuret of ammonia.

### Resolutions of the Dock Jennerian Society.

The undersigned medical gentlemen of this town have seen with extreme concern the late malignancy of the Small-pox, which, in several instances, have again swept away nearly whole families. They lament these effects of this destructive disease the more at this time, because *they have immediately arisen from the introduction of it by inoculation*; a practice which, from its very commencement, has, by maintaining a source of perpetual circulation to this fatal poison, added considerably to its general mortality. Adverting to these irresistable facts, they have again consulted together; and, after the most deliberate re-consideration of all the existing circumstances connected with Vaccine Inoculation, feel themselves *conscientiously* and *professionally* called upon to adhere to their former resolutions and reciprocal engagements; and have accordingly renewed their determination, *not to inoculate for the Small-pox*, excepting after the inoculation for the Cow-pox; and that only in peculiar circumstances, where an experiment may be required to satisfy doubting parents. But in no instance can they *recommend* the exposure to so *malignant a poison.*

They judge the communication of these Resolutions, coupled with the practice of inoculating their *own families with the cow-pock exclusively*, the most decisive and satisfactory means of conveying to the public their perfect reliance on this mild preventive, for protection against the small-pox.

While the undersigned are industriously and disinterestedly endeavouring to remove *so pestilential a disease* from this town, it would give them the highest satisfaction to see the medical gentlemen in the neighbouring towns, generally and unitedly engaged in adopting similar means, to accomplish so desirable an end.

In prosecuting, therefore, the attainment of a great public benefit, by a means sanctioned and recommended by the Legislature, they deem it no deviation from the strictest attention to *professional etiquette*, respectfully to solicit the co-operation of their medical brethren.

Vaughan May, M. D.

Robert Sargent,

Daniel Little,

Richard Dunning,

Digory Morris Spry,

John Smith,

John Lower,

John Penkivil,

John Bone.

N. B. The Subscribers again invite the poor in this neighbourhood to avail themselves of this mild and effectual protection against the small-pox; they can be inoculated gratuitously at the usual time and place, or at any time, by application to the respective houses of the subscribers.

*Plymouth, Dec.* 14, 1804.

BILL

### *BILL OF MORTALITY,*

### FOR PORTSMOUTH, NEW HAMPSHIRE, for A. D. 1803.

### BY LYMAN SPALDING, M.B. &c.

| COMPLAINT. | AGE. | Jan. | Feb. | Mar. | Apr. | May | June | July | Aug. | Sept | Oct. | Nov. | Dec. | Total. |
|---|---|---|---|---|---|---|---|---|---|---|---|---|---|---|
| Abscess in the lungs | 19, 53 years | | 1 | | | | | | | | 1 | | | 2 |
| Apoplexy | 85, 54, 48 years | | | 1 | | 1 | | | | | 1 | | | 3 |
| Asthma | 40 years | | | | 1 | | | | | | | | | 1 |
| Atrophy | 2, 12, 2m. 11d. 3m. 2y. 18, 14m. 3w. | 1 | | | | | 1 | 1 | 1 | 1 | 2 | | 2 | 9 |
| Bleeding from the lungs | 55, 61 years | | | | 1 | | 1 | | | | | | | 2 |
| Cankerrash or Scarlatina | 11, 6m. 1m. 5, 1, 17, 2, 25, 22, 35, 1, 20, 3, 3, 22, 11, 6, 4, 10, 3m. 6, 10m. 9, 37, 3, 3, 4m. 4, 12, 16, 1 years | 6 | 5 | 5 | | | 2 | 4 | 5 | 2 | 2 | | | 31 |
| Cholera of Infants | 3y. 6m. 18 months | | | | | | | 1 | | 1 | 1 | | | 3 |
| Cholic bilious | 33, 30 years | 1 | | | | | | 1 | | | | | | 2 |
| Consumption | 40, 69, 39, 38, 25, 36, 24, 18, 27, 72, 43, 38, 35, 48, 15, 22, 24, 15, 44, 60 years | 4 | 2 | 4 | | | 2 | 1 | | 1 | 2 | | 4 | 20 |
| Convulsions | 60, 20, 2w. 3w. 1w. 4w. 38, 1d. 14, 1, 39 years | | | 1 | 1 | | 2 | 3 | 2 | | | | 2 | 11 |
| Dropsy | 55, 40, 28, 3, 47, 77, 40, 44, 22 years | 2 | | 2 | | | | 2 | 1 | | 1 | | 1 | 9 |
| Dropsy in the brain | 10, 4m. 18, 6m. | 2 | | | | | 1 | | | | | 1 | | 4 |
| Dropsy on the head | 2 years | | | | | 1 | | | | | | | | 1 |
| Dropsy in the breast | 21 years | | | | | | | | 1 | | | | | 1 |
| Dysentery | 9 months | | | | | | | | | 1 | | | | 1 |

| | | | | | | | | | | | | | | |
|---|---|---|---|---|---|---|---|---|---|---|---|---|---|---|
| Epilepsy | 40, 1 years | | | | | | | | | | 1 | | 1 | 2 |
| Erysipelas | 5 months | | | | 1 | | | | | | | | | 1 |
| Fever and Ague | 18 years | | | | | 1 | | | | | | | | 1 |
| Fever bilious | 17, 40 years | | | 1 | | 1 | | | | | | | | 2 |
| Fever pulmonic | 50, 22, 46, 50, 72, 23, 62, 56, 70, 72, 7, 77, 35 | | | 1 | 2 | 5 | 2 | | | 1 | 1 | 1 | | 13 |
| Fever typhus | 71, 53 years | | | 1 | | | | | | | | 1 | | 2 |
| Inflammation of the bowels | 23 years | | | | | | 1 | | | | | | | 1 |
| Jaundice | 49 years | | 1 | | | | | | | | | | | 1 |
| Lock-Jaw | 38 years | | 1 | | | | | | | | | | | 1 |
| Nephritis | 43 years | | | | | | | 1 | | | | | | 1 |
| Old age | 87, 88, 74, 80, 74, 82, 78, 78, 86, 86 years | 3 | 1 | 2 | | 1 | 1 | 2 | | | | | | 10 |
| Palsy | 58, 82, 36 years | | | | 1 | 1 | | | | | | 1 | | 3 |
| Phrenzy | 48, 24 years | | | | | | 1 | | | | | 1 | | 2 |
| Premature birth | | | | | 1 | | | | 1 | | | | | 2 |
| Quinsy | 2, 22 years | | | | | | | | | | 1 | 1 | | 2 |
| Scrophula | 12 years | | | 1 | | | | | | | | | | 1 |
| *Casualties.* Overlaid | 1 month | | | | 1 | | | | | | | | | 1 |
| *Casualties.* Suicide | 28 years | | | | | | | | 1 | | | | | 1 |
| Total | | 19 | 11 | 19 | 9 | 11 | 14 | 16 | 12 | 7 | 13 | 6 | 10 | 147 |

BIRTHS. Males – 106, Females – 107; Total – 213

Portsmouth, situated 43°, 5′ North; 6°, 26′ East Longitude from Washington, contains about 6000 inhabitants.

### Royal Jennerian Society.

Dr. Walker, in a letter to the Editors, dated Salisbury-square, 3, xij, 1804, says,—"The notice which ye gave last summer (1804) has brought an unremitting demand on the Central House for the matter of inoculation from all parts of the empire; and the applicants have not yet been disappointed. Will ye request them to send their letters, under cover, to Francis Freeling, Esq. General Post Office. I have been struck with the delicacy of those gentlemen who have left the enclosed letters unsealed. Such proof to the Postmasters General, though not required by them, shews that no private business is carried on by abuse of their generous liberality; shews that the humane and patriotic object, of giving facility to the Society, and to those who are disposed to co-operate with it in the pious attempt to exterminate the small-pox, is not perverted. In one or two instances, medical men remain unsupplied, from their giving their address imperfectly; some such cause is the only one that can ever put them into such predicament, and possibly excite in them a suspicion of neglect.

"Those who receive matter from the Central House ought to be in possession of the information that it is mostly sent to them on the day on which it was taken, which is commonly the viij. day of inoculation. At an earlier period it is peculiarly active, but small in quantity; at a later, it is less to be depended upon. This latter description of it I sometimes use myself, knowing that I shall have the opportunity of seeing the effect of it, and of re-inoculating in case of failure; but I should badly merit the confidence of the Medical Council of the Society, if I sent out matter to the world of which I was not certain of its producing the genuine vacciola.

---

### St. Thomas's and Guy's Hospitals.

The Lectures for the ensuing Season, at these adjoining Hospitals, will commence on the 1st of February.

*St. Thomas's Hospital.*—Anatomy, human and comparative, by Mr. Cline and Mr. Cooper. Principles and Practice of Surgery, by Mr. Cooper.

*Guy's Hospital.*—Practice of Medicine, by Dr. Babington and Dr. Curry.

Chemistry and Experimental Philosophy, by Dr. Babington and Mr. Allen.

Theory of Medicine and Materia Medica, by Dr. Curry.

Midwifery and the Diseases of Women and Children, by Dr. Haighton.

Physiology, or Laws of the Animal Economy, by Dr. Haighton.

Regular Clinical Lectures on select Medical Cases, by Drs. Babington, Curry, and Marcet.

Some time in the Spring will be given a Course of Lectures on the Structure and Diseases of the Teeth, by Mr. Fox, surgeon-dentist; and on the Veterinary Art, by Mr. Coleman, Professor at the Veterinary College.

These several Lectures are so arranged, that no two of them interfere with each other in the hours of attendance; and the whole is calculated to form a complete Course of Medical and Chirurgical Instruction. Terms, and other particulars, to be learnt from Mr. Stocker, apothecary to Guy's Hospital, who is also empowered to enter gentlemen as pupils to such of the lectures as are delivered at Guy's. Among the numerous improvements that are now carrying on at Guy's Hospital, intended to facilitate and to increase the acquisition of medical and chirurgical science to pupils, not only a new, enlarged, and commodious Lecturing Theatre has been built, so contrived as to admit of the several chemical processes being carried on before the class; but there is already in considerable forwardness, an extensive Library and Museum, in which, along with the valuable collection of books belonging to the Physical Society, there will be deposited the various morbid preparations that are daily accumulating, with a correct account of the respective cases to which they belonged, so as to form a registry of Practice, and a collection of facts, that must prove of the utmost importance to future inquirers.

Numbers of gentlemen in extensive business, to whom opportunities occur of procuring preparations of singular rarity and value, but which become nearly useless when confined to their small private collections, will thus have the means not only of rendering them extensively beneficial, but of perpetuating their names as contributors to the common stock of Medical Science. Accordingly, not only those who have received their professional education at this Hospital, but many others, whose names and talents are of the first rank, have promised their assistance towards the undertaking.

---

*Medical Theatre, St. Bartholemew's Hospital.*

The Spring Courses of Lectures will begin at this Theatre in the following order:

On the Theory and Practice of Medicine, by Dr. Roberts and Dr. Powell.

On Anatomy and Physiology, by Mr. Abernethy.

On the Theory and Practice of Surgery, by Mr. Abernethy.

On Comparative Anatomy and the Laws of Organic Existence, by Mr. Macartney.

On Chemistry and the Materia Medica, by Dr. Powell and Dr. Edwards.

On Midwifery and the Diseases of Women and Children, by Dr. Thynne.

The Anatomical Lectures will begin about the 30th of January, 1805, and the other Lectures in the course of the same week.

Further particulars may be known by applying to Mr. Nicholson, at the apothecary's shop, St. Bartholemew's Hospital.

---

*Theatre, London Hospital.*

Mr. Headington and Mr. Frampton, will commence their Spring Course of Lectures upon Anatomy, Physiology, and the Principles and Operations of Surgery, on Saturday, January 19, at two o'clock.

Demonstrations and Dissection as usual, by Mr. Armiger.

Dr. Dennison will lecture on the Theory and Practice of Midwifery.

*Medical Theatre, Clifford Street, Burlington Gardens.*

Dr. BADHAM's Course of Lectures on Medicine and Chemistry, will commence on Monday, January 21, at the usual hour. The Medical Lectures at eight, the Chemical at nine in the morning; a Clinical Lecture will be delivered twice a week, on such cases as may be sufficiently interesting in the limits of the Westminster General Dispensary. For further particulars apply to Dr. Badham, No. 15, Clifford Street, where, or at the Dispensary in Gerrard Street, a prospectus may be had.

*Theatre of Anatomy, Blenheim Street, Great Marlborough Street.*

Mr. BROOKES will commence his Spring Course of Lectures on Anatomy, Physiology, and Surgery, on Saturday, January 19, at two o'clock. A suite of commodious apartments, thoroughly ventilated, and replete with every convenience for the purposes of dissecting and injecting, will be open every morning till two o'clock, where Mr. Brookes attends.

Dr. BRADLEY's Lectures on the Practice of Medicine will commence on Wednesday, the 9th of January next, at his house, No. 25, Parliament Street.

Dr. BATTY will commence his Spring Course of Lectures on the Theory and Practice of Midwifery, and on the Diseases of Women and Children, on Monday, January 21, at half past ten o'clock, at his house, Great Marlborough Street.

Mr. BLAIR's Physiological Lectures, for the information of scientific and professional gentlemen, amateurs of Natural History, students in the liberal and fine Arts, &c. will recommence on the 8th of January; and be continued every succeeding Tuesday and Friday evening, at eight o'clock precisely. This Course is illustrated by select anatomical preparations, drawings, models, casts, and a living muscular subject. Further particulars may be learnt at Mr. Blair's house, Great Russel-street, Bloomsbury, where may be had a printed Syllabus of the Lectures.

Mr. CHEVALIER will commence his next Course of Lectures on the Principles and Operations of Surgery, on Wednesday, Jan. 30, at seven o'clock in the evening, at his house, South Audley Street, Grosvenor Square, where printed particulars may be had.

We are happy to learn, that the Proposal laid before the Public several months since by Mr. SANDERS, for the Establishment of a Dispensary for the Relief of Diseases of the EYE and EAR, continues to meet with the most respectable patronage; and that the fund already subscribed is sufficient to warrant the immediate organization of the Charity.

